# Daytime Performance in Insomnia Patients

**DOI:** 10.1111/jsr.70234

**Published:** 2025-10-30

**Authors:** Sibylle Frase, Katharina Domschke, Bernd Feige, Jonas Hosp, Claas Lahmann, Kai Spiegelhalder, Derek Spieler, Dieter Riemann, Lukas Frase

**Affiliations:** ^1^ Department of Neurology and Neuroscience Medical Center ‐ University of Freiburg, Faculty of Medicine, University of Freiburg Freiburg Germany; ^2^ Department of Psychiatry and Psychotherapy Medical Center ‐ University of Freiburg, Faculty of Medicine, University of Freiburg Freiburg Germany; ^3^ Department of Psychosomatic Medicine and Psychotherapy Medical Center ‐ University of Freiburg, Faculty of Medicine, University of Freiburg Freiburg Germany

**Keywords:** alertness, cognitive functioning, daytime functioning, fatigue, sleep, sleepiness, vigilance

## Abstract

Patients suffering from Nonorganic Insomnia (NI) are burdened by significant subjective daytime impairments which contribute to the reduction of quality of life for the patients and lead to greater healthcare utilization and increased indirect costs. Besides being central to diagnosis, operationalized criteria for daytime symptoms are lacking and data remain heterogeneous, especially regarding objectively measurable deficits. This study examines daytime performance in 329 NI patients through neuropsychological testing as well as self‐report questionnaires, and correlates the results with polysomnographic data. In the main analysis, neuropsychological data, normalized for age and health status, displayed no impairment of vigilance or alertness—contrary to what is often assumed for NI and what would typically be expected in cases of sleepiness. For secondary analyses, neuropsychological data was then correlated with self‐report and polysomnographic measures, and comparisons between NI patients with and without comorbidities were conducted. NI patients displayed a positive correlation of performance with nocturnal arousal markers, predominantly during REM sleep and a slightly diminished capability to increase focus in the phasic compared to tonic alertness paradigm. In summary, the current study in a well characterized large sample of NI patients with state‐of‐the‐art measures of the most sensitive markers for sleep related daytime impairment found no evidence for diminished general vigilance or alertness due to sleep loss. The results help to understand conflicting evidence on neurocognitive deficits in insomnia by distinguishing between alertness or vigilance deficits and subtle changes in neurocognitive processing that might be better interpreted in line with underlying hyperarousal and anxiety.

**Abbreviations**
AI: arousal index
ESS: Epworth Sleepiness Scale
FSS: Fatigue Severity Scale
ISI: Insomnia Severity Index
NI: Nonorganic Insomnia
(N)REM: (non) rapid eye movement
PSQI: Pittsburgh Sleep Quality Index
REML: REM sleep latency
SDB: sleep‐disordered breathing
SEI: sleep efficiency
SOL: sleep onset latency
SWS: slow wave sleep
TAP: test of attentional performance
TST: total sleep time
WASO: wake time after sleep onset

## 1 Introduction

Difficulties initiating or maintaining sleep burden as much as 30%–50% of the general population, while 10% fulfill the complete diagnostic criteria for Nonorganic Insomnia according to ICD‐10 (NI (Morin et al. 2006; Perlis et al. 2022; World Health Organization 2016)). Insomnia increases the risk for depression (Baglioni et al. 2011) and decreases the quality of life in affected individuals (Kyle et al. 2010; Léger et al. 2001). Moderate to severe insomnia is associated with greater healthcare utilization and increased indirect costs compared to good sleeping controls (Sarsour et al. 2011). A large part of these indirect costs is incurred from loss of productivity and increased absenteeism at work (Daley et al. 2009; Sivertsen et al. 2006). In line with the impact of insomnia on daytime functioning, the presence of subjective daytime symptoms such as fatigue, decreased energy, daytime sleepiness, mood disturbance and cognitive impairment, is considered a diagnostic prerequisite (World Health Organization 2016). Daytime impairment is usually assessed via self‐report questionnaires, but operationalized diagnostic criteria for daytime symptoms are missing (Riemann et al. 2015). Therefore, diagnostic evaluation of daytime impairments remains a challenge. In their newest edition, the Diagnostic and Statistical Manual of Mental Disorders (5th ed.; DSM‐5; (American Psychiatric Association 2013)), broadly formulates insomnia disorder to cause “clinically significant distress or impairment in social, occupational, educational, academic, behavioural or other important areas of functioning.” Defining insomnia‐specific daytime symptoms is further complicated by high levels of symptom overlap with frequent comorbid disorders such as depression (Riemann et al. 2024) or sleep‐disordered breathing (Lee and Ahn 2025). Either daytime symptoms of (unknown) comorbid disorders appear insomnia‐related, or the impact of insomnia gets underrated while focusing on the comorbid disorder. In addition, a bidirectional interaction between disorders can influence the perceived severity of daytime symptoms (Riemann et al. 2024).

In Germany, the medico‐legal requirement to assess fitness to drive in some patients with sleep disorders has led to the proposal of a standardized approach on assessing sleep‐related daytime impairment (Bundesanstalt für Straßenwesen, Bergisch Gladbach 2022). The proposed neuropsychological testing comprises different domains of alertness and vigilance (Bundesanstalt für Straßenwesen, Bergisch Gladbach 2022; Frase et al. 2020). Alertness is conceptualized as the general state of central nervous system activation that allows an individual to react to its environment, making it the basis for every attentional performance. Alertness can be further sub‐categorized into tonic and phasic alertness, the first describing an individual's intrinsic, “baseline” alertness, the latter describing a targeted arousal during limited periods in response to a stimulus (that precedes the target of an attentional task in an experimental setting).

Vigilance describes a form of sustained attention, where the attentional focus is actively maintained over a prolonged period (Mirsky et al. 1991). From the current literature, it remains inconclusive whether vigilance is impaired in insomnia disorder. While some studies report decreased speed and accuracy in insomnia patients conducting vigilance tasks (Hauri 1997; Perrier et al. 2015; Varkevisser and Kerkhof 2005), others did not find group differences in comparison to participants with undisturbed sleep (Orff et al. 2007). Daytime impairment appears associated with objectively measured sleep (Orff et al. 2007), however data on correlations between subjectively reported severity of daytime impairment and neuropsychological measures remain inconclusive. A recent meta‐analysis concludes insomnia to be associated with poorer cognitive performance overall and in several specific subdomains, but it also highlights the high level of heterogeneity across studies (Wardle‐Pinkston et al. 2019). The authors define three characteristics future studies should adhere to in order to improve data quality: application of operationalized diagnostic criteria for insomnia, usage of appropriate assessment measures and examination of nocturnal sleep prior to neurocognitive testing (Wardle‐Pinkston et al. 2019).

In line, this study applies a specific neuropsychological test battery proposed to discriminate sleep‐related difficulties in daytime functioning (Test of Attentional Performance, TAP (Zimmermann and Fimm 2007)) and includes only patients who were diagnosed by an experienced psychiatrist following routine in‐patient sleep diagnostics in a specialized psychiatric sleep laboratory. The results were normalized for age and health status and then analyzed to characterize insomnia‐related daytime impairment in a representative clinical population with or without comorbidities. In addition, correlations between the assessed measures and standard sleep and vigilance‐related self‐report questionnaires as well as objective nocturnal sleep parameters during the night prior to testing were examined.

## 2 Methods

### 2.1 Study Population

Between June 1st, 2018, and May 31st, 2023, patients undergoing polysomnography in the sleep laboratory of the Department of Psychiatry and Psychotherapy, Medical Center—University of Freiburg, Germany, were assessed for objective deficits in alertness and vigilance as part of the routine diagnostic procedure. All patients underwent prior out‐patient care and in‐patient diagnostics/treatment due to the necessity of polysomnography following a standard stepped care pathway according to Frase et al. (2020). All patients were asked to undergo the examination without concurring medication if possible. If medical treatment was needed, patients were asked to refrain from changing medication at least for 2 weeks prior to the sleep laboratory visit and did not use on‐demand medication during the visit.

In total, 329 individual patient cases could be detected where patients underwent neuropsychological testing as part of their inpatient diagnostic routine in the sleep laboratory and were diagnosed as suffering from NI according to ICD‐10 (World Health Organization 2016). One hundred (out of 329) NI patients were diagnosed with NI only; in 229 cases additional, partly overlapping comorbid disorders were identified as potentially relevant for the sleep complaints. It is to note, that patients were screened as part of the clinical pathway and only referred to this sleep laboratory if they did not express a high pre‐test probability for sleep‐disordered breathing (SDB) or suffered from remaining sleep complaints under successful SDB treatment.

Table 1 displays the diagnosed comorbidities of NI patients (individual diagnoses were aggregated as diagnostic groups; patients who received more than one relevant diagnosis are listed in each respective group).

**TABLE 1:** Comorbidities of Nonorganic Insomnia patients (*n* = 329).

Comorbidities	*N*	Age (year) ± SD	Gender (m/f/d)
No relevant comorbidities	100	46.7 ± 15.6	39/61/0
Sleep disordered breathing	64	55.0 ± 12.0	37/27/0
Other sleep disorders	84	52.6 ± 14.4	29/55/0
Affective disorders	124	45.6 ± 14.6	55/69/0
Post traumatic disorders	5	32.0 ± 7.9	1/4/0
Other mental disorders	28	39.1 ± 15.7	13/15/0
Neurological disorders	11	56.0 ± 13.5	3/8/0
Somatic disorders	18	53.3 ± 14.3	4/14/0

For some analyses, slightly different subsamples were analyzed as the tasks were standardized for different age ranges (alertness: 19–89 years; vigilance: 20–69 years).

### 2.2 Study Design

All measurements were conducted as part of routine clinical diagnostics at the Department of Psychiatry and Psychotherapy, Medical Center—University of Freiburg, Germany. Patients stayed for two to four nights in the sleep laboratory (depending on the specific clinical questions), with neuropsychological testing typically performed on the morning following the first night of polysomnographic recording. Patients were given the opportunity to shower and have a light breakfast prior to testing. The study was conducted in accordance with the Declaration of Helsinki, approved by the Ethics Committee of the Medical Center—University of Freiburg (23‐1384‐S1‐retro), and registered in the German Register for Clinical Studies (www.germanctr.de, DRKS00032531).

### 2.3 Neuropsychology

Insomnia‐related daytime performance was characterized via the *Alertness* and *Vigilance* subtests of the computer‐based *Test of Attentional Performance* (TAP, Zimmermann and Fimm 2007).

The *Alertness* subtest examines the intensity of attention under two conditions. The first condition assesses simple reaction time to a visual cue: a cross appears on the monitor, and the participant is instructed to press a key as quickly as possible. For the second condition, the stimulus is preceded by a warning tone. This set‐up allows for differentiating between baseline levels of alertness (“tonic arousal”) and temporal orientation of attentional focus (“phasic arousal”). The test duration spans across 5 min with repeated measurements of both conditions. Reaction time, measured in milliseconds, is defined as the main outcome parameter and can be compared between the two conditions. The subtest has been normalized for participants aged 19–89 years (Bodenburg et al. 2001; Zimmermann and Fimm 2007).

The *Vigilance* subtest measures the capability of long‐term maintenance of attention in a setting characterized by low stimulus density. Participants are instructed to focus on two differently pitched auditory stimuli. As the baseline pattern, the two tones alternate, but sometimes, in changing intervals, two tones with the same pitch follow each other. Participants should react to these repetitions of tones by pressing a key as quickly as possible. The monotonous task spans 30 min, thereby allowing an evaluation of monotony tolerance and the capability to sustain attention over a long period. The subtest has been normalized for participants aged 20–69 years (Zimmermann and Fimm 2007).

### 2.4 Self‐Report Questionnaires

The *Insomnia Severity Index* (ISI, Morin et al. 2011) is a 7‐item measure that evaluates insomnia symptoms on a scale from 0 (none) to 4 (very severe). The questionnaire is considered state of the art for the evaluation of insomnia severity with the total score interpreted as follows: 0–7: no insomnia, 8–14: subthreshold insomnia, 15–21: moderate clinical insomnia, 22–28: severe clinical insomnia. One item specifically measures the interference of sleep problems with daily functioning including fatigue, mood, daily work/chores, memory and mood.

The *Pittsburgh Sleep Quality Index* (PSQI, Buysse et al. 1989) was designed to measure sleep quality in clinical populations. The questionnaire comprises 19 items that can be used to aggregate seven subscores and one total score. One of the subscores was created to estimate daytime dysfunction. The global PSQI score can discriminate between good (< 6 points) and disturbed sleep (> 5 points).

Daytime sleepiness was assessed via the *Epworth Sleepiness Scale* (ESS, Johns 1991). Participants rate their tendency to become sleepy between 0 (no chance of dozing) and 3 (high chance of dozing) for eight common daytime activities. Total sum score can be interpreted as follows: 0–7: low daytime sleepiness, 8–9: average daytime sleepiness, 10–15: potential excessive daytime sleepiness, 16–24: excessive daytime sleepiness.

The *Fatigue Severity Scale* (FSS, Krupp et al. 1989) was used to evaluate the impact of fatigue on patients in the past week. Participants rate the impact of fatigue on nine dimensions of their day‐to‐day activity between 1 (strong disagreement with statement) and 7 (strong agreement with statement). Either the total sum score (suggested cut‐off: 36 points) or the mean score can be interpreted. Expectable means for several disorders are published and can be used for comparison (e.g., patients with multiple sclerosis: 5.1 points).

### 2.5 Polysomnography

Polysomnography was recorded from 11:00 PM to 7:00 AM according to standard procedures as described previously (e.g., Frase et al. 2023). All recordings included an EEG (C3‐A2, analogue filter setting 0.53–70 Hz, sampling rate 200 Hz), electrooculogram, submental electromyogram and an electrocardiogram. Polysomnographic recordings were visually scored off‐line according to standard criteria by experienced raters who were blind to the experimental conditions (Berry et al. 2020).

The following polysomnographic parameters of sleep continuity and architecture were assessed: sleep onset latency (SOL), defined as the time between turning the lights off and the first 30 s epoch of stage 2 sleep (N2), slow wave sleep (SWS/N3) or rapid eye movement (REM) sleep; total sleep time (TST), defined as the time spent in stage 1 or 2 sleep, slow wave sleep (SWS) or REM sleep; sleep efficiency (SEI), defined as the ratio of TST to time in bed × 100%; wake after sleep onset (WASO), defined as the time spent awake between sleep onset and final awakening; number of wake periods; arousal index (AI), defined as the number of arousals per hour of sleep for TST; percentages of stage 2 sleep, SWS and REM sleep referred to TST; REM sleep latency (REML), defined as the period between sleep onset and the occurrence of the first 30 s epoch of REM sleep.

### 2.6 Statistical Analyses

All neuropsychological outcome variables were investigated with repeated‐measures analyses of variance (ANOVA) with either the within‐subject factor condition (tonic alertness, phasic alertness) and/or the between‐subject factor group (with or without comorbidities). As no control group of healthy participants could be included from the data set, percent ranks normalized for age and health status as provided by the automatic evaluation included in the respective measures were used to detect effects of insomnia on the outcome measures (Zimmermann and Fimm 2007).

All self‐report and polysomnography‐derived outcome variables were investigated with age‐corrected repeated‐measures analyses of variance (ANOVA) with the between‐subject factor group (with or without comorbidities). Exploratory correlation analyses were conducted to explore the relationship between the three outcome domains (neuropsychology, self‐reports, polysomnography).

In cases of violations of sphericity, the Greenhouse–Geisser adjustment was applied. The level of significance was set at *p* < 0.05 (two‐tailed). Descriptive values are given as means and standard deviations. For the estimation of effect sizes, partial η^2^ values were calculated (*p*η^2^; small: < 0.06; medium: ≥ 0.06 and < 0.14; large: ≥ 0.14). All analyses were conducted with the statistical software IBM SPSS Statistics (Version 29).

## 3 Main Results

### 3.1 Neuropsychology—*Alertness*

**FIGURE 1:**
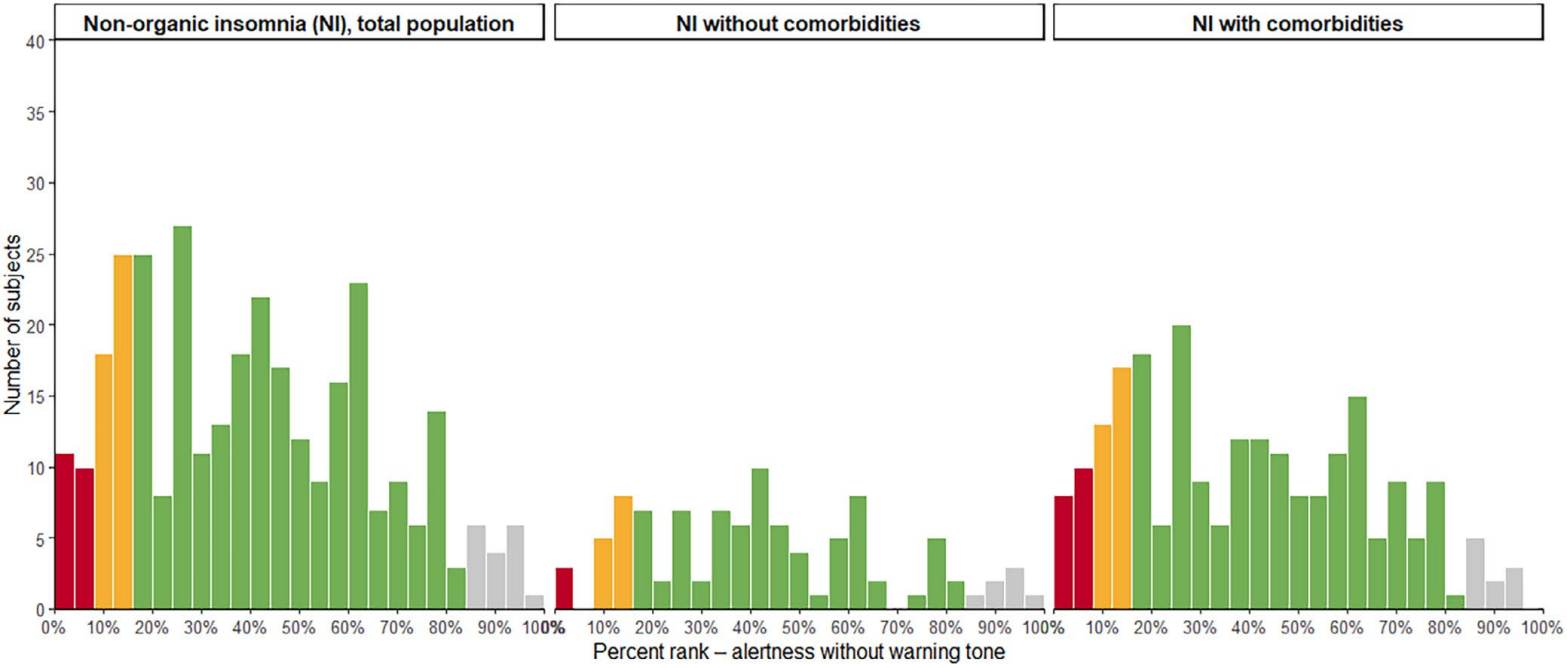
Number of subjects for each reaction time percent rank in the tonic alertness paradigm (without warning tone). Panel 1: Total population of patients with non‐organic insomnia (NI). Panel 2: Subgroup of NI patients without comorbidities. Panel 3: Subgroup of NI patients with comorbidities. Green bars, normal reaction time percent ranks (16–83); yellow bars, medium impaired reaction time percent ranks (American Psychiatric Association 2013; Bundesanstalt für Straßenwesen, Bergisch Gladbach 2022; Daley et al. 2009; Frase et al. 2020; Lee and Ahn 2025; Riemann et al. 2015, 2024; Sivertsen et al. 2006); red bars, severely impaired reaction time percent ranks (0–7).

**FIGURE 2:**
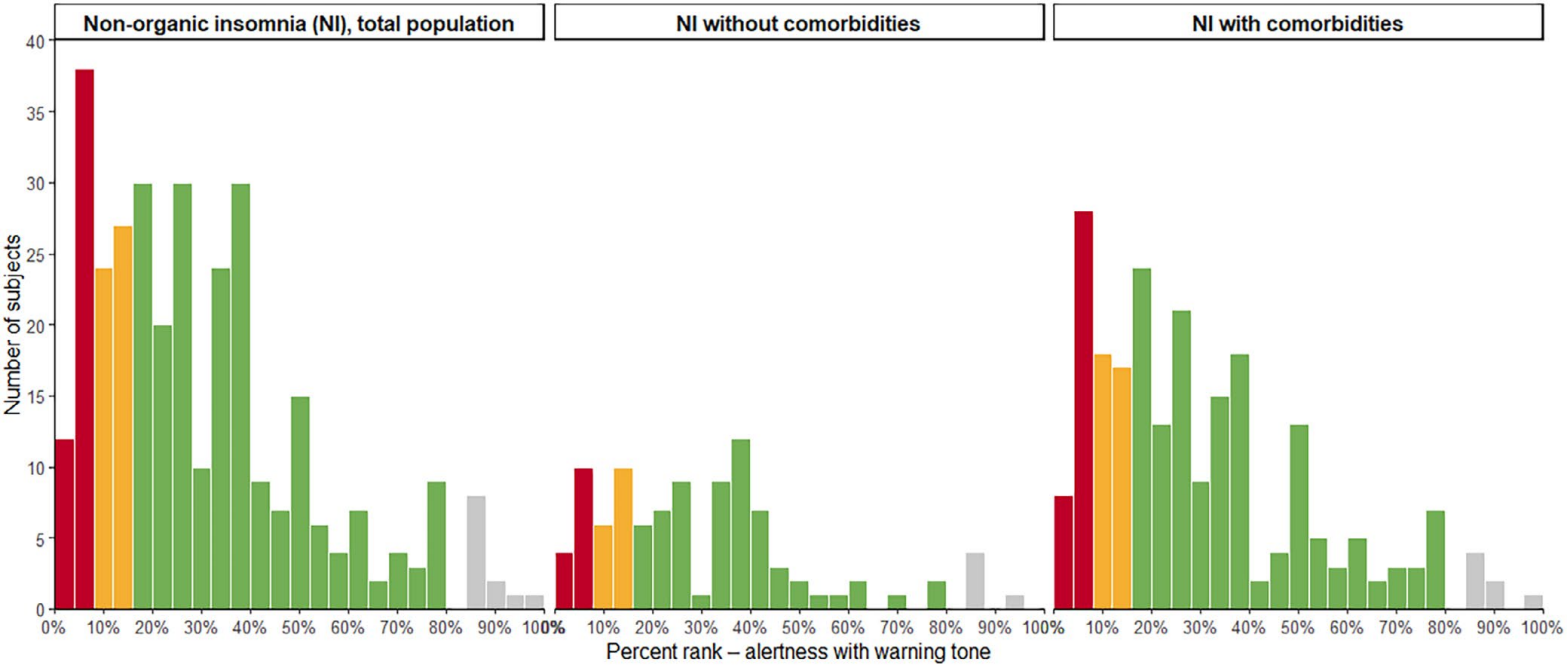
Number of subjects for each reaction time percent rank in the phasic alertness paradigm (with warning tone). Panel 1: Total population of patients with non‐organic insomnia (NI). Panel 2: Subgroup of NI patients without comorbidities. Panel 3: Subgroup of NI patients with comorbidities. Green bars, normal reaction time percent ranks (16–83); yellow bars, medium impaired reaction time percent ranks (American Psychiatric Association 2013; Bundesanstalt für Straßenwesen, Bergisch Gladbach 2022; Daley et al. 2009; Frase et al. 2020; Lee and Ahn 2025; Riemann et al. 2015, 2024; Sivertsen et al. 2006); red bars, severely impaired reaction time percent ranks (0–7).

In total, NI patients displayed normal overall reaction times around 270 ms which represents a percent rank of ~40% and can be interpreted as undisturbed. Figure one and two display the range and total number of percent ranks reached in the tonic (without warning tone, Figure 1) and phasic (with warning tone, Figure 2) alertness paradigm. While most patients showed reaction times in the expected normal range (green bars, percent ranks 16–83), a small number of NI patients displayed medium (yellow bars, percent rank 8–15) or severely diminished alertness (red bars, percent rank 0–7). It is to note, that most cases with reduced reaction times were part of the subgroup with comorbidities.

When changing from the tonic to the phasic alertness paradigm, an improvement of reaction time in response to the warning tone of around 14 ms would be expected. In contrast, mean reaction times did not differ between tonic and phasic alertness (*Δ* = + 2.1 ms, *F* = 1.0, *p* = 0.324, *pη* ^2^ = 0.003, Table 2A). This lack of improvement led to a highly significant percent rank shift of 10 points between tonic and phasic alertness for NI patients (*F* = 157.6, *p* < 0.001, *pη* ^2^ = 0.330, Table 2A).

**TABLE 2:** Reaction times of Nonorganic Onsomnia patients with and without comorbidities during tonic and phasic alertness.

		Range (min–max)
(A) NI patients (*n* = 329)		
Tonic alertness		
Reaction time, mean ± SD	271.1 ± 65.4 ms	190–710 ms
Reaction time, median ± SD	263.7 ± 62.8 ms	185–715 ms
Reaction time, percent rank ± SD	39.7% ± 24.7%	1%–96%
Phasic alertness		
Reaction time, mean ± SD	273.2 ± 61.7 ms	186–690 ms
Reaction time, median ± SD	266.4 ± 61.8 ms	180–707 ms
Reaction time, percent rank ± SD	29.6% ± 22.1%	1%–98%
(B) NI patients without comorbidities (*n* = 100)		
Tonic alertness		
Reaction time, mean ± SD	263.0 ± 54.8 ms	191–510 ms
Reaction time, median ± SD	256.5 ± 53.7 ms	185–510 ms
Reaction time, percent rank ± SD	41.9% ± 24.8%	1%–96%
Phasic alertness		
Reaction time, mean ± SD	270.7 ± 59.5 ms	186–541 ms
Reaction time, median ± SD	263.3 ± 58.2 ms	181–540 ms
Reaction time, percent rank ± SD	30.1% ± 21.6%	1%–92%

To examine the influence of comorbidities on these values, we then analyzed the subgroup of 100 NI patients without comorbidities. These patients showed the same pattern of results, with reaction times around 267 ms and no improvement in the test set‐up for phasic alertness (*Δ* = + 7.7 ms, *F* = < 0.1, *p* = 0.870, *pη* ^2^ = < 0.001, Table 2B). Again, this lack of improvement led to a highly significant percent rank shift of 12 points between tonic and phasic alertness for NI patients without comorbidities (*F* = 74.2, *p* < 0.001, *pη* ^2^ = 0.433, Table 2B).

### 3.2 Neuropsychology—*Vigilance*

In total, NI patients displayed undisturbed reaction times around 630 ms, which fall into the middle of the normal range. Interestingly, NI patients without comorbidities showed significantly quicker reaction times (*Δ* = −26 ms, *F* = 4.5, *p* = 0.034, *pη* ^2^ = 0.014; age corrected) with a smaller standard deviation and smaller maximum values (Table 3).

**TABLE 3:** Reaction times of Nonorganic Insomnia patients with and without comorbidities during prolonged vigilance.

Vigilance		Range (min–max)
(A) NI patients (*n* = 325)		
Reaction time, mean ± SD	632.0 ± 139.9 ms	399–1613 ms
Reaction time, median ± SD	618.3 ± 136.2 ms	394–1585 ms
Reaction time, percent rank ± SD	43.4% ± 24.6%	0%–96%
Number of errors, mean ± SD	3.7 ± 9.1	0–137
Number of errors, percent rank ± SD	54.1% ± 25.4%	0%–82%
Number of missings, mean ± SD	1.0 ± 1.9	0–14
Number of missings, percent rank ± SD	29.3% ± 18.2%	1%–50%
(B) NI patients without comorbidities (*n* = 99)		
Reaction time, mean ± SD	606.6 ± 88.7 ms	428–888 ms
Reaction time, median ± SD	594.7 ± 86.0 ms	427–884 ms
Reaction time, percent rank ± SD	45.1% ± 21.1%	5%–90%
Number of errors, mean ± SD	2.5 ± 2.9	0–20
Number of errors, percent rank ± SD	57.2% ± 22.1%	4%–82%
Number of missings, mean ± SD	0.9 ± 2.0	0–14
Number of missings, percent rank ± SD	33.9% ± 20.4%	1%–50%

Both groups displayed relatively few numbers of errors and nonresponses with mean values in the expected normal range, with NI patients without comorbidities displaying a smaller standard deviation and value range regarding the number of errors (Table 3).

## 4 Secondary Analyses

### 4.1 Self‐Report Questionnaires

On average, patients reported moderate clinical insomnia with a total ISI score of 16.5 and significantly disturbed sleep with 11.7 points in the PSQI (see Table 4). Insomnia patients with comorbidities reported slightly higher levels of disturbed sleep in the PSQI (*Δ* = 1.1, *F* = 4.5, *p* = 0.035, *pη* ^2^ = 0.015). No differences in insomnia severity could be detected between patients with and without comorbidities (ISI; *Δ* = 0.9, *F* = 2.7, *p* = 0.101, *pη* ^2^ = 0.009).

**TABLE 4:** Self‐report measures on sleep and daytime symptoms of Nonorganic Insomnia patients with and without comorbidities.

		Range (min–max)
(A) NI patients		
ISI (*N* = 308)		
Total sum score, mean ± SD	16.5 ± 4.6	3–28
Daytime impairment, mean ± SD	2.7 ± 1.0	0–4
PSQI (*N* = 300)		
Total sum score, mean ± SD	11.7 ± 3.8	3–20
Daytime dysfunction, mean ± SD	1.4 ± 0.7	0–3
ESS (*N* = 311)		
Total sum score, mean ± SD	7.1 ± 5.0	0–22
FSS (*N* = 306)		
Total sum ± SD	40.9 ± 14.0	9–63
Mean score ± SD	4.5 ± 1.6	1–7
(B) NI patients without comorbidities		
ISI (*N* = 97)		
Total sum score, mean ± SD	15.8 ± 4.3	4–28
Daytime impairment, mean ± SD	2.6 ± 0.9	0–4
PSQI (*N* = 96)		
Total sum score, mean ± SD	11.0 ± 3.7	3–18
Daytime dysfunction, mean ± SD	1.2 ± 0.6	0–2
ESS (*N* = 97)		
Total sum score, mean ± SD	6.8 ± 5.1	0–19
FSS (*N* = 97)		
Total sum ± SD	37.7 ± 14.3	9–63
Mean score ± SD	4.2 ± 1.6	1–7

Regarding daytime impairment, the respective subscale of the PSQI detected small but significant differences between patients with and without comorbidities (*Δ* = 0.3, *F* = 16.3, *p* = < 0.001, *pη* ^2^ = 0.051), while the corresponding subscale of the ISI did not (*Δ* = 0.2, *F* = 1.5, *p* = 0.226, *pη* ^2^ = 0.005). No significant group differences could be found for daytime sleepiness in the ESS (*Δ* = 0.4, *F* = 0.4, *p* = 0.497, *pη* ^2^ = 0.002), but fatigue appeared higher in patients with comorbidities (FSS total sum score: *Δ* = 4.7, *F* = 9.5, *p* = 0.002, *pη* ^2^ = 0.030; mean score: *Δ* = 0.5, *F* = 9.5, *p* = 0.002, *pη* ^2^ = 0.030). In summary, patients with comorbidities displayed slightly higher values of disturbed sleep (PSQI), daytime impairment (PSQI) and fatigue (FSS), but did not differ from patients without comorbidities in the ISI or ESS. No significant correlations between TAP measures and self‐report measures were detected besides a minor correlation between fatigue scores (FSS) and *Vigilance* (*r* = 0.100/0.103; see Table S1).

### 4.2 Polysomnography

Overall, NI patients displayed disturbed nocturnal sleep with a relevant WASO of 77.5 min as well as a slightly prolonged SOL of 33.4 min. No significant differences between patients with and without relevant comorbidities could be detected (all *p* > 0.05, data not shown). Table 5 provides further details on sleep continuity and architecture.

**TABLE 5:** Polysomnographic data of Nonorganic Insomnia patients with and without comorbidities prior to neuropsychological testing.

		Range (min–max)
(A) NI patients (*N* = 296)		
Sleep continuity		
SOL, min ± SD	33.4 ± 33.0	2–277
TST, min ± SD	356.4 ± 68.2	49–467.5
SEI, % ± SD	74.2 ± 14.2	10.2–95.8
WASO, min ± SD	77.5 ± 46.1	12–260
Wake periods ± SD	31.8 ± 12.5	6–74
AI (TST), 1/h ± SD	20.0 ± 8.8	3.1–71.5
AI (REM), 1/h ± SD	27.8 ± 12.9	0–82.1
Sleep architecture		
NREM Stage 2, %TST ± SD	61.7 ± 9.2	32.8–85.0
SWS, %TST ± SD	8.8 ± 9.4	0–50.3
REM, %TST ± SD	18.5 ± 6.4	0–39.1
REML, min ± SD	118.4 ± 70.7	0–403
(B) NI patients without comorbidities (*N* = 91)		
Sleep continuity		
SOL, min ± SD	38.8 ± 45.5	3–277
TST, min ± SD	357.5 ± 73.7	49–448.5
SEI, % ± SD	74.4 ± 15.4	10.2–94.6
WASO, min ± SD	77.5 ± 46.1	15–219.5
Wake periods ± SD	30.0 ± 11.6	6–71
AI (TST) ± SD	19.7 ± 9.2	3.1–52.4
AI (REM), 1/h ± SD	27.7 ± 12.7	5.7–73.2
Sleep architecture		
NREM Stage 2, %TST ± SD	61.7 ± 9.0	32.8–80.7
SWS, %TST ± SD	8.9 ± 9.4	0–50.3
REM, %TST ± SD	18.8 ± 6.2	0–31.7
REML, min ± SD	105.8 ± 63.7	0–380

**FIGURE 3:**
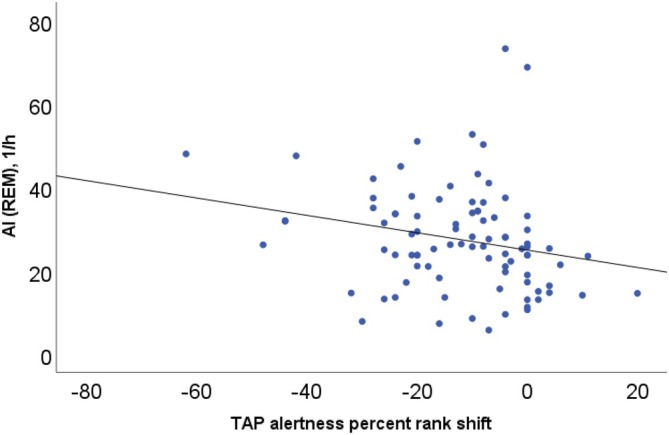
Correlation analysis of the arousal index during REM sleep and the percent rank shift between tonic and phasic alertness reaction times of Nonorganic Insomnia patients. Patients with higher arousal indices, predominantly during REM sleep, displayed lesser capability to improve their reaction times after prompting (negative percent rank shift; *r* = −0.228).

Regarding the relationship between nocturnal sleep and daytime functioning, a pattern of correlations could be detected. Higher numbers of wake periods as well as a higher arousal index correlated with better tonic and phasic *Alertness* reaction times, while lower percentages of slow wave sleep and stage 2 NREM sleep (as well as a longer REM latency) correlated with worse reaction times (NI patients without comorbidities, Table 6). In contrast to the better absolute performance in NI patients with higher arousal indices, REM and overall AI correlated with a stronger negative percent rank shift (NI patients without comorbidities, Table 6 and Figure 3). In summary, patients with higher arousal indices, predominantly during REM sleep, displayed a quicker general tonic and phasic alertness reaction time, but lesser capability to improve their reaction times after prompting (difference between phasic and tonic alertness/% rank shift). Regarding *Vigilance*, higher percentage of stage 2 NREM sleep and a loner REM sleep latency correlated with better reaction times (NI patients without comorbidities, Table 6). NI patients with comorbidities displayed a similar, but less pronounced pattern (supplements, Table S2).

**TABLE 6:** Correlation analyses of polysomnographic and neuropsychological variables of Nonorganic Insomnia patients without comorbidities.

	*r*	*N*	Lower CI	Upper CI
Phasic alertness reaction time (percent rank)				
SOL	−0.025	90	−0.231	0.183
TST	−0.032	90	−0.238	0.176
SEI	−0.033	90	−0.238	0.175
WASO	0.030	90	−0.178	0.236
Wake periods	0.370	90	0.177	0.536
AI (TST)	0.258	90	0.053	0.441
AI (REM)	0.321	89	0.121	0.496
%NREM 2	−0.174	90	−0.368	0.034
%SWS	−0.160	90	−0.355	0.049
%REM	−0.050	90	−0.254	0.159
REML	−0.146	89	−0.344	0.064
Tonic alertness reaction time (percent rank)				
SOL	−0.037	91	−0.241	0.170
TST	−0.026	91	−0.231	0.181
SEI	−0.026	91	−0.230	0.181
WASO	0.025	91	−0.182	0.230
Wake periods	0.333	91	0.137	0.505
AI (TST)	0.286	91	0.085	0.465
AI (REM)	0.356	90	0.161	0.505
%NREM 2	−0.107	91	−0.306	0.102
%SWS	−0.172	91	−0.365	0.035
%REM	−0.071	91	−0.273	0.136
REML	−0.138	90	−0.335	0.071
Alertness percent rank shift				
SOL	0.041	90	−0.168	0.246
TST	0.006	90	−0.201	0.213
SEI	0.006	90	−0.202	0.212
WASO	−0.034	90	−0.239	0.174
Wake periods	−0.094	90	−0.295	0.115
AI (TST)	−0.177	90	−0.370	0.032
AI (REM)	−0.228	89	−0.417	−0.021
%NREM 2	−0.050	90	−0.255	0.158
%SWS	0.034	90	−0.174	0.240
%REM	0.060	90	−0.149	0.264
REML	−0.006	89	−0.214	0.203
Vigilance reaction time (percent rank)				
SOL	−0.022	81	−0.239	0.197
TST	−0.011	81	−0.229	0.208
SEI	−0.017	81	−0.235	0.202
WASO	0.037	81	−0.138	0.253
Wake periods	−0.010	81	−0.228	0.209
AI (TST)	0.030	81	−0.189	0.247
AI (REM)	−0.001	80	−0.221	0.219
%NREM 2	0.114	81	−0.107	0.325
%SWS	−0.057	81	−0.272	0.163
%REM	−0.031	81	−0.247	0.189
REML	0.114	80	−0.109	0.325

## 5 Discussion

As the main finding in this very well characterized large sample of NI patients with state‐of‐the‐art measures of the most sensitive markers for sleep related daytime impairment, the study found no evidence for diminished general vigilance or alertness due to sleep loss. The findings support a rising number of studies failing to demonstrate objectively measured sleep loss to be the main factor diminishing daytime performance in insomnia besides the common conception of insomnia being determined by sleep loss and its consequences. Instead, a characterization of insomnia symptoms including subjective daytime impairments as mostly independent of objective sleep physiology is more in line with the pathophysiological understanding of anxiety or somatoform disorders.

Attentional deficits in general are commonly reported daytime symptoms in insomnia and are the most extensively studied cognitive domain in insomnia (Brownlow et al. 2020; Spiegelhalder et al. 2010). However, comparability across studies is limited since the subdomains of attention that were assessed varied (i.e., focused, sustained or shift attention, (Shekleton et al. 2010)). In general, there is considerable inhomogeneity across studies regarding inclusion criteria and neuropsychological test measures, which, together with small sample sizes, may have limited statistical power and masked attentional deficits (Brownlow et al. 2020). The lack of consistent neuropsychological findings (Fortier‐Brochu et al. 2012) has contributed to the perception that daytime symptoms might be a consequence of attention bias towards sleep‐related activities rather than actual cognitive impairments (Harvey 2002; Spiegelhalder et al. 2008). The current study adds a neurobiological interpretation based on the hyperarousal model of insomnia (Dressle and Riemann 2023; Riemann et al. 2010).

Current concepts define hyperarousal as a state of increased cognitive, emotional, physiological and cortical arousal across 24 h which significantly contributes to the experienced insomnia symptoms (Dressle and Riemann 2023).

In this study, performance in the alertness paradigm was positively correlated with nocturnal arousal markers, predominantly during REM sleep. The capability to increase focus in the phasic alertness paradigm compared to tonic levels was, however, significantly diminished and correlated negatively with the same arousal markers. For basic attention processes, increased arousal appears beneficial, which might explain the initially surprising findings of normal basic reaction times and similar neurocognitive functions in patients with insomnia (Altena et al. 2008; Edinger et al. 1997; Horne 2010; Kyle et al. 2017; Shekleton et al. 2014) and represents another differentiation to patients suffering from objective sleep disturbances such as sleep disordered breathing.

It has been argued that cognitive deficits in insomnia disorder are subtle and can therefore only be observed in more complex cognitive tasks. In an exemplary study in 76 insomnia patients, switching attention and working memory were impaired while simple or complex sustained attention tasks were unremarkable (Shekleton et al. 2014). The current study can be interpreted in line with these findings by demonstrating a disturbed capability to increase focus when changing from the simple tonic alertness task to the more complex phasic alertness paradigm. The negative correlation between this capability and nocturnal arousal markers strengthens the concept of a bi‐directional influence of (hyper‐)arousal in insomnia, where basic neurocognitive tasks remain undisturbed while more difficult or complex tasks de‐mask specific impairments (Cellini et al. 2014; Edinger et al. 2013). Interestingly, the strongest correlation could be detected with the amount of arousals during REM sleep. The finding adds to the growing body of evidence for the high importance of REM sleep (micro‐)arousal for the pathophysiological understanding of insomnia (Benz et al. 2020; Feige et al. 2018, 2023). The proposed characterization and interpretation of daytime impairment in NI patients being predominantly influenced not by sleep loss, but by increased arousal supports a potential conceptualization of NI as sharing similarities with anxiety or somatoform disorders rather than other sleep disorders such as sleep disordered breathing.

Vigilance was chosen as the second examined neurocognitive domain, due to its high clinical and safety relevance. In summary, insomnia patients demonstrated normal reaction times without any relevant restraint and reported no excessive daytime sleepiness in the ESS. Interestingly, NI patients without comorbidities showed significantly quicker reaction times with a smaller standard deviation and smaller maximum values and comprised no individuals with abnormally high numbers of errors, again leading to a smaller standard deviation and value range. This finding further supports the main finding of undisturbed vigilance functions in patients with (nonorganic) insomnia and the interpretation of vigilance deficits to be mostly attributable to comorbid disorders such as sleep‐disordered breathing.

In line, insomnia patients with comorbidities reported slightly higher values of disturbed sleep (PSQI), daytime impairment (PSQI) and fatigue (FSS). Fatigue has sometimes been conceptualized in differentiation to sleepiness/vigilance and comprises a broader concept of subjective arousal and energy deficits during the day. The current study, for the first time, reports representative values for insomnia patients using the FSS. Here, patients reported fatigue levels between 4.2 and 4.5 depending on comorbidities. While the FSS is not normalized, reference values exist for healthy participants without fatigue (2.8) as well as several conditions that typically are accompanied by relevant fatigue such as autoimmune and/or neurological disorders (e.g., systemic lupus erythematosus: 4.6, multiple sclerosis: 5.1 (Krupp et al. 1989)). This comparison highlights insomnia as a disorder with clinically relevant levels of fatigue that need to be better addressed in day‐to‐day clinical care.

The findings on vigilance and fatigue underline the importance to discriminate between subjective daytime impairment and daytime sleepiness. While all NI patients suffer from some form of subjective daytime impairment by definition, daytime sleepiness/disruption of vigilance is not typical and should be treated as a red flag for another or comorbid disorder.

As a major strength, the study analyzes a robust real world data set with subjective and objective sleep continuity and architecture measures as well as subjective and objective scores of daytime impairment. Included NI patients have been carefully diagnosed according to standard criteria and express subjective and objective parameters that can be considered very representative for the disorder, with a relevant burden of disease depicted in the self‐report measures (ISI, PSQI) as well as polysomnography (WASO, SOL, TST). Overall, the study design fully adheres to quality criteria recently proposed for examinations of daytime symptoms in insomnia (Wardle‐Pinkston et al. 2019). The large study population allows for a distinction between the broader sample of insomnia patients with comorbidities and a clear defined subgroup of patients suffering only from insomnia without any comorbidities.

Some effect sizes of the reported correlation analyses are medium to small, so results should be interpreted with care. Replication studies or further investigation of the main results should be conducted before firm clinical applications can be derived. While the tasks were chosen based on existing diagnostic guidelines for the evaluation of sleep‐related daytime symptoms (Bundesanstalt für Straßenwesen, Bergisch Gladbach 2022), further studies should focus on the insomnia‐specific lack of active‐focus‐related task improvement. In addition, longer task duration up to several hours might be needed to detect a diminution of hyperarousal based performance gains and a stronger reflection of insomnia‐related neurocognitive deficits (Shekleton et al. 2014).

In a clinical setting, the resulting influences on specific aspects of daytime functioning are often mixed between beneficial and disrupting effects of hyperarousal as well as impairment due to comorbidity and acute or chronic sleep loss. This further complicates objective assessment of daytime impairment in insomnia.

## 6 Conclusions

As the main finding in this very well characterized large sample of NI patients with state‐of‐the‐art measures of sleep related daytime impairment, the study found no evidence for diminished general vigilance or alertness due to sleep loss. The results support an interpretation of NI as a disorder sharing similar characteristics with anxiety or somatoform disorders rather than other sleep disorders such as sleep disordered breathing. The occurrence of severe objectively measurable deficits such as daytime sleepiness appears not typical for NI patients and should be treated as a red flag for another or a comorbid disorder.

From a clinical perspective, the importance of a quick and reliable assessment of daytime functioning in insomnia remains high, but the practical application is difficult. As a secondary finding, insomnia might be accompanied by good or even improved functioning in basic neurocognitive tasks such as ‘alertness’ due to increased levels of baseline arousal on one hand and constraints to further raise cognitive arousal when needed for specific tasks, for example, focus/’phasic alertness' on the other. This might explain conflicting evidence on whether there are objective neurocognitive deficits in insomnia at all.

NI patients appear to suffer from levels of daytime fatigue comparable to chronic autoimmune disorders, but do not display relevantly restricted vigilance functions. The results underline the importance to discriminate between subjective daytime fatigue and daytime sleepiness and to treat patients accordingly.

## Author Contributions

Conceptualization, Supervision, Resources: K.D., C.L., J.H., D.R., L.F. Investigation, formal analysis, data curation: S.F., B.F., L.F. Validation and Methodology: S.F., B.F., J.H., K.S., D.S., L.F. Writing – original draft, Visualization: S.F., L.F. Writing – review and editing: K.D., B.F., J.H., C.L., K.S., D.S., D.R. All authors provided final approval and agreed to be accountable for all aspects of the work.

## Ethics Statement

The study was conducted in accordance with the Declaration of Helsinki and was approved by the Ethics Committee of the University Medical Center Freiburg (23‐1384‐S1‐retro), and registered in the German Register for Clinical Studies (www.germanctr.de, DRKS00032531).

## Consent

The authors have nothing to report.

## Conflicts of Interest

K.D. is a member of the Neurotorium Editorial Board, Lundbeck Foundation. All other authors declare that they have no competing interests.

## Supporting information


**TABLE S1:** Correlation analyses of self‐report and neuropsychological variables.

**TABLE S2:** Correlation analyses of polysomnographic and neuropsychological variables of Nonorganic Insomnia patients with comorbidities.

## Data Availability

The data that support the findings of this study are available on request from the corresponding author. The data are not publicly available due to privacy or ethical restrictions.
