# Supplementary material for: Daytime Performance in Insomnia Patients
Source: J Sleep Res. 2025 Oct 30;35(3):e70234. doi: 10.1111/jsr.70234 (PMC13193394; doi:10.1111/jsr.70234)
Supplement: Supplementary file 1 — TABLE S1: Correlation analyses of self‐report and neuropsychological variables. [file JSR-35-e70234-s002.docx]

**Tab. S1:**

|  |  | r | N | Lower C.I. | Upper C.I. |
| --- | --- | --- | --- | --- | --- |
| *Phasic alertness, reaction time (percent rank)* | ESS, total sum score | -,005 | 305 | -,117 | ,108 |
|  | FSS, total sum score | -,070 | 300 | -,181 | ,044 |
|  | FSS, mean score | -,072 | 300 | -,184 | ,042 |
|  | ISI, daytime impairment | -,003 | 302 | -,116 | ,110 |
|  | PSQI, daytime dysfuntion | -,081 | 298 | -,192 | ,033 |
| *Tonic alertness reaction time (percent rank)* | ESS, total sum score | -,023 | 305 | -,135 | ,089 |
|  | FSS, total sum score | -,084 | 301 | -,195 | ,029 |
|  | FSS, mean score | -,087 | 301 | -,198 | ,026 |
|  | ISI, daytime impairment | -,029 | 302 | -,142 | ,084 |
|  | PSQI, daytime dysfuntion | -,056 | 299 | -,169 | ,058 |
| Vigilance, mean reaction time | ESS, total sum score | ,071 | 307 | -,041 | ,181 |
|  | FSS, total sum score | ,100 | 302 | -,013 | ,211 |
|  | FSS, mean score | ,103 | 302 | -,010 | ,213 |
|  | ISI, daytime impairment | ,042 | 304 | -,071 | ,154 |
|  | PSQI, daytime dysfuntion | ,075 | 300 | -,039 | ,186 |
