# Supplementary material for: Daytime Performance in Insomnia Patients
Source: J Sleep Res. 2025 Oct 30;35(3):e70234. doi: 10.1111/jsr.70234 (PMC13193394; doi:10.1111/jsr.70234)
Supplement: Supplementary file 2 — TABLE S2: Correlation analyses of polysomnographic and neuropsychological variables of Nonorganic Insomnia patients with comorbidities. [file JSR-35-e70234-s001.docx]

**Tab. S2:**

|  |  | r | N | Lower CI | Upper CI |
| --- | --- | --- | --- | --- | --- |
| Phasic alertness. reaction time (percent rank) | SOL | .010 | 291 | -.105 | .125 |
|  | TST | -.019 | 291 | -.134 | .096 |
|  | SEI | -.015 | 291 | -.129 | .101 |
|  | WASO | .029 | 291 | -.086 | .143 |
|  | Wake periods | .130 | 291 | .015 | .241 |
|  | AI (TST) | .156 | 291 | .042 | .267 |
|  | AI (REM) | .078 | 290 | -.038 | .191 |
|  | %NREM 2 | -.069 | 291 | -.182 | .046 |
|  | %SWS | -.135 | 291 | -.246 | -.021 |
|  | %REM | .040 | 291 | -.076 | .154 |
|  | REML | .116 | 290 | .001 | .229 |
| Tonic alertness reaction time (percent rank) | SOL | -.009 | 291 | -.123 | .107 |
|  | TST | .065 | 291 | -.051 | .178 |
|  | SEI | .067 | 291 | -.048 | .181 |
|  | WASO | -.066 | 291 | -.179 | .050 |
|  | Wake periods | .086 | 291 | -.029 | .199 |
|  | AI (TST) | .137 | 291 | .023 | .248 |
|  | AI (REM) | .100 | 289 | -.015 | .213 |
|  | %NREM 2 | -.067 | 291 | -.181 | .048 |
|  | %SWS | -.116 | 291 | -.228 | -.001 |
|  | %REM | .077 | 291 | -.038 | .190 |
|  | REML | .028 | 289 | -.087 | .143 |
| Alertness percent rank shift | SOL | .009 | 289 | -.194 | .035 |
|  | TST | -.104 | 289 | -.217 | .012 |
|  | SEI | -.100 | 289 | -.213 | .015 |
|  | WASO | .118 | 289 | .003 | .231 |
|  | Wake periods | .043 | 289 | -.073 | .157 |
|  | AI (TST) | -.081 | 289 | -.194 | .035 |
|  | AI (REM) | -.054 | 288 | -.168 | .062 |
|  | %NREM 2 | .018 | 289 | -.098 | .133 |
|  | %SWS | -.008 | 289 | -.123 | .108 |
|  | %REM | -.044 | 289 | -.159 | .071 |
|  | REML | .115 | 288 | -.001 | .227 |
| Vigilance. mean reaction time | SOL | .012 | 292 | -.103 | .127 |
|  | TST | .030 | 292 | -.085 | .145 |
|  | SEI | .033 | 292 | -.082 | .147 |
|  | WASO | -.104 | 292 | -.216 | .011 |
|  | Wake periods | -.001 | 292 | -.116 | .114 |
|  | AI (TST) | -.023 | 292 | -.137 | .092 |
|  | AI (REM) | -.032 | 290 | -.147 | .083 |
|  | %NREM 2 | .056 | 292 | -.060 | .169 |
|  | %SWS | -.004 | 292 | -.119 | .111 |
|  | %REM | -.042 | 292 | -.156 | .073 |
|  | REML | -.083 | 290 | -.196 | .032 |
